# Impact of Delayed Early Antiretroviral Therapy Initiation on Treatment Outcomes in Infant Macaques Exposed to SHIVAD8

**DOI:** 10.3390/v17060849

**Published:** 2025-06-14

**Authors:** Li Ma, Yoshiaki Nishimura, Xueling Wu, Olivia Donau, Eunice Vincent, Hong Lu, Robert V. Blair, Lara A. Doyle-Meyers, Malcolm Martin, Ronald S. Veazey, Huanbin Xu, Xiaolei Wang

**Affiliations:** 1 Tulane National Primate Research Center, Tulane University School of Medicine, 18703 Three Rivers Road, Covington, LA 70433, USA; lma3@tulane.edu (L.M.);; 2 Laboratory of Molecular Microbiology, National Institute of Allergy and Infectious Diseases, National Institutes of Health, Bethesda, MD 20892, USA; ynishimura@niaid.nih.gov (Y.N.);; 3 Department of Medicine, Infectious Diseases, Boston University School of Medicine, Boston Medical Center, 650 Albany Street, Boston, MA 02118, USA; xueling.wu@bmc.org (X.W.);

**Keywords:** HIV/SIV, infants, early antiretroviral therapy, viral remission

## Abstract

Infants born to HIV-positive mothers remain at significant risk of HIV acquisition despite maternal adherence to antiretroviral therapy, cesarean delivery, and formula feeding. Our previous study reported that initiating early antiretroviral treatment at three days post-SIV infection resulted in approximately eighty percent of pediatric virologic remission. In this study, we investigated treatment outcomes in postnatally SHIV-exposed infant macaques when early intervention was delayed by two days, as well as the mechanisms underlying virologic control. The results showed that, although initiating treatment at five days post-exposure effectively suppressed viral replication, only one of the three infant macaques achieved a sustained state of virologic remission following analytical treatment interruption. Notably, this virus-controlled infant lacked detectable virus-specific immunity, including neutralizing antibodies, cytotoxic T cell responses, and antibody-dependent cellular cytotoxicity. These findings highlight the critical importance of early treatment initiation as a key determinant of virologic control in HIV-exposed, infected infants. This study provides valuable insights for guiding early pediatric HIV intervention strategies in clinical settings.

## 1. Introduction

The rapid establishment of viral reservoirs harboring replication-competent virus remains the major obstacle to achieving an HIV functional cure or cure in adults. In adult macaques intrarectally infected with SIV/SHIV, early initiation of combination antiretroviral therapy (cART), even within days of infection, has failed to produce curative outcomes [1,2,3]. These findings indicate that viral reservoirs are rapidly seeded in adults post HIV/SIV infection, posing a significant challenge to efforts aimed at achieving ART-free virologic remission or cure. Despite significant progress in preventing mother-to-child transmission, pediatric HIV infections still primarily occur during labor and delivery through newborn exposure to the virus. Globally, approximately 150,000 infants and children are newly infected with HIV each year (UNAIDS, 2023) [4], and without treatment, these children are at high risk of rapidly progressing to AIDS-related illnesses. Importantly, the susceptibility to HIV and immune responses in developing infants differ markedly from those in adults [5,6,7]. Notably, children living with HIV often develop broadly neutralizing antibodies (bnAbs) more rapidly than adults, likely due to the presence of a distinct population of bnAb-producing B cell precursors [8,9]. While immediate initiation of ART may restrict viral reservoir size [10,11,12,13,14,15,16,17], there have been only a few reported cases of drug-free pediatric HIV remission, including those documented in the Pediatric AIDS Clinical Trials [6,11,17,18,19,20,21,22,23,24]. This limited success may be partly attributed to treatment regimens commonly used in HIV-exposed infants, which typically combine dual nucleoside reverse transcriptase inhibitors (NRTIs) with a protease inhibitor, often without incorporating an integrase strand transfer inhibitor (INSTI), such as dolutegravir (DTG) [14,15,17,20,25,26,27,28,29]. Other contributing factors include limitations in sample collection that hinder comprehensive assessment of viral reservoirs [19,20,24,26,29,30,31] and the risk of viral rebound following discontinuation or interruption of lifelong daily treatment, whether due to noncompliance or other challenges [14,24,26,30,32,33,34,35].

Utilizing the pediatric NHP model of HIV exposure, previous studies have shown that cell-associated viral RNA/DNA can be detected in certain tissues during the very early days of infection [36,37,38,39,40,41,42,43,44]. Our recent studies demonstrate that proviral reservoir seeding in neonatal macaques is not fully established by 3 days following intravenous (IV) SIV inoculation at birth, even though both plasma viral load and cellular SIV RNA/DNA are readily detectable as early as 1 day post inoculation (dpi). Interestingly, our results show that initiating a short-term early cART (FTC/TFV/DTG) for 28 days at 3, 4, or 5 dpi leads to markedly different dynamics of plasma viral load. When treatment is initiated at 3 dpi, the plasma viral load becomes undetectable by 7 dpi and remains so thereafter. In contrast, even a one- or two-day delay in treatment initiation (at 4 or 5 dpi) results in persistent viremia. Furthermore, a 9-month course of daily cART, initiated at 3 dpi, led to ART-free viral remission in 4 out of 5 infant macaques infected with SIV within 24 h of birth [45]. These findings suggest that the precise timing of early intervention prevention is critical for reducing viral reservoir size and effectively controlling viral replication in HIV/SIV-exposed infected infants [46,47].

In this study, we further investigated the clinical outcomes of SHIV-exposed, infected infant macaques that received the same 9-month daily cART regimen, but with a two-day treatment delay in treatment initiation, starting at 5 days post-SHIV infection. We also assessed whether prolonged exposure to the SHIV envelope, resulting from delayed treatment, could induce the development of neutralizing antibodies. The CCR5-tropic simian-human immunodeficiency virus (SHIV) AD8 strain efficiently infects rhesus macaques, leading to persistent high-level viremia. SHIV AD8 is highly pathogenic, causing substantial CD4^+^ T cell depletion and progression to AIDS within two years in some macaques [48,49,50]. Notably, infection with SHIV AD8 can elicit broadly neutralizing antibodies (bnAbs) against the HIV Env in individual macaques, which has been associated with partial viral control [51,52,53,54]. In contrast, SIVmac infection typically induces a delayed and low-titer neutralizing antibody response and is generally resistant to bnAb induction [55,56]. In this study, one of three infants did not exhibit viral rebound after cessation of treatment. However, this virus-controlled infant macaque lacked detectable immune correlates of protection, including virus-specific neutralizing antibodies, cytotoxic CD8+ T responses, and antibody-dependent cellular cytotoxicity. These findings highlight the importance of early treatment initiation and suggest that virological factors may play a pivotal role in achieving a sustained state of ART-free viral remission in HIV-exposed, infected infants.

## 2. Materials and Methods

### 2.1. Ethics Statement

All animals in this study were housed at the Tulane National Primate Research Center in accordance with the Association for Assessment and Accreditation of Laboratory Animal Care International standards. All studies were reviewed and approved by the Tulane University Institutional Animal Care and Use Committee. Animal housing and studies were carried out in strict accordance with the recommendations in the Guide for the Care and Use of Laboratory Animals of the National Institutes of Health (NIH, AAALAC #000594). All clinical procedures were carried out under the direction of a laboratory animal veterinarian. All procedures were performed under anesthesia using ketamine+/− dexdormitor/antisedan or tiletamine/zolazepam, and analgesics, including buprenorphine, were provided for any potentially painful procedure performed. All efforts were made to minimize stress, improve housing conditions, and provide enrichment opportunities (e.g., objects to manipulate in the cage, varied food supplements, foraging and task-oriented feeding methods, interaction with caregivers and research staff).

### 2.2. Animals and Simian-Human Immunodeficiency Virus

A total of 6 newborn, Indian-origin rhesus macaques (*Macaca mulatta*, RMs) with random sex difference were used in this study. Newborn macaques were intravenously inoculated with 300 TCID50 CCR5-tropic SHIV AD8 on the day of birth (within 24 h), as previously described [49]. To assess the impact of delayed treatment, three infant macaques received combination antiretroviral therapy (cART) consisting of three anti-HIV drugs: tenofovir (TFV, 20 mg/kg/day), emtricitabine (FTC, 30 mg/kg/day), and dolutegravir (DTG, 2.5 mg/kg/day) administered subcutaneously. Treatment began at 5 days post-infection and continued daily for up to 9 months. An additional three untreated infant animals served as controls. Blood samples were collected weekly to monthly, while LN and rectal biopsies were obtained bi-monthly or post-ATI. Plasma and single-cell suspensions were prepared to analyze plasma viral load and cell-associated DNA. Virus-controlled infant animal at month 14 after analytic treatment interruption received a single high-dose challenge with SHIV AD8 via the intrarectal route as described [57]. The challenge virus stock was diluted in a total volume of 1 mL serum-free RPMI 1640 medium, equivalent to 1000TCID50. An animal was considered infected if their plasma viral loads exceeded 50 copies/mL for 2 consecutive weeks.

### 2.3. Tissue Collection and Phenotyping

PBMC were isolated from EDTA-treated venous blood by density gradient centrifugation with Lymphocyte Separation Medium (Fisher Scientific, Houston, TX, USA). Tissue lymphocytes were isolated from the LN and rectal tissue biopsies as described [58]. Briefly, LNs were minced, passed through nylon mesh screens, and washed twice in RPMI containing 5% fetal calf serum. The rectal biopsy was incubated with RPMI containing EDTA for 30 m with shaking at 37 °C, followed by collagenase digestions (two 30-min intervals) and processed into a single cell by a blunt syringe needle. Lymphocytes isolated were used to extract cellular DNA/RNA or stained for flow cytometry analysis, as we previously reported [45,58]. Cells were stained with: CD3 (SP34), CD4 (L200), CD8 (SK1), CD28 (28.2), CD95 (DX2), and LIVE/DEAD Fixable Aqua Dead Cell Stain Kit (Invitrogen, Grand Island, NY). Isotype-matched controls were included in all experiments. All antibodies and reagents were purchased from BD Biosciences Pharmingen (San Diego, CA, USA). Samples were resuspended in BD Stabilizing Fixative (BD Biosciences) and acquired on a FACS FORTESSA (Becton Dickinson, San Jose, CA, USA). Data was analyzed with FlowJo software (Version 10.8.1, Tree Star, Ashland, OR, USA).

### 2.4. Nucleic Acid Extraction

Fresh PBMCs or tissue-derived lymphocytes were processed to extract cellular genomic DNA and cellular RNA using the AllPrep DNA/RNA Mini Kit (Cat No: 80311, Qiagen, Germantown, MD, USA) according to the manufacturer’s instructions. The extracted cellular DNA and RNA samples were stored at −80 °C for qPCR analysis.

### 2.5. Quantification of Plasma Viral Load and Cell-Associated SHIVAD8 DNA

Specific primer sets and probes were synthesized by integrated DNA technologies and used to measure plasma viral load and CA SIV DNA, as seen in Supplementary Table S2. Plasma viral load targeting SIV gag is used to determine plasma viral load with a limit of detection of 50 copies per mL. Plasma viral RNA was extracted by QIAamp Viral RNA Mini kit (Qiagen, Hilden, Germany), the extracted RNA was reverse transcribed into cDNA using a ThermoFisher SuperScript™ VILO™ Master Mix (Invitrogen, Grand Island, NY, USA) a thermocycler at 25 °C for 10 min and 42 °C for 60 min, followed by an enzyme inactivation step at 85 °C for 5 min. cDNA was further used to quantify total SIV transcripts by digital PCR (Applied Biosystems QuantStudio Absolute Q Digital PCR System, Thermo Fisher, USA). QuantStudio™ Absolute Q™ MAP16 Plate Kit and Master Mix (Thermo Fisher, USA) were used. The cycling conditions were: 10 min at 96 °C, 40 cycles of 96 °C/15 s and 60 °C/30 s. The SIV gag primer/probe set was shown in Table S2. Copies of SIV DNA, expressed as copies per one million cells, were measured and normalized to cellular input, as determined by copies of genomic RPPR30 (two copies of rhesus macaque RPPR30 DNA per cell). Quantification of total SIV DNA, which was isolated from a minimum of 2 × 10^5^ cell equivalents for individual samples, was expressed as copies per 1 million cells (LOD, 5 copies per one million cell equivalents).

### 2.6. SIV Neutralization Antibody Assay

SIV neutralization was measured using single-round infection of TZM-bl cells with Env pseudoviruses [58]. Briefly, 40 μL of virus was incubated for 30 min at 37 °C with 10 μL of serially diluted plasma in duplicate wells before the addition of TZM-bl cells. To maintain consistent assay conditions, sham medium was used in place of plasma in specified control wells. Plasma dilutions were defined at the point of incubation with virus supernatant. Virus infection levels were determined after 2 days by a luciferase assay (Promega, Madison, WI, USA). Neutralization curves were fitted using the 5-parameter nonlinear regression function built into Prism 9.0 (GraphPad Prism 9 Software, La Jolla, CA, USA), and 50% inhibitory dilutions (ID_50_) were defined as the plasma reciprocal dilutions required to inhibit viral infection by 50%.

### 2.7. SIV Gag-Specific CD8+ T Cell Assay

To detect SIV gag-specific CD8^+^ T-cell responses as we previously described [59,60], PBMCs were stimulated by a pool of 15-mer gag peptides (5 µg/mL each peptide), medium (negative control), or phorbol-12-myristate-13-acetate (PMA; 5 ng/mL; MilliporeSigma, Burlington, MA, USA) plus ionomycin (50 µg/mL) (positive control) with or without MVC treatment (10 µM; NIH) for 6 h. The cultures also contained brefeldin A (MilliporeSigma) and 1 µg/mL of anti-CD49d and anti-CD28 costimulatory molecules (BD Biosciences). Cultured cells were stained with monoclonal antibodies specific for surface molecules (CD3, CD4, and CD8) and a Live/Dead cell-staining kit. After fixation and permeabilization with Cytofix/Cytoperm solution (BD Biosciences), cells were stained with antibodies specific for IFN-γ and TNF-α and then washed with Perm/Wash buffer (BD Biosciences). Finally, labeled cells were fixed in 1.5% paraformaldehyde, acquired with a FORTESSA cytometer, and data were analyzed using FlowJo^TM^ v10 software. Only samples in which the percentage of cytokine-stained cells was at least twice that of background were considered positive.

### 2.8. In Vivo CD8+ Lymphocyte Depletion

To deplete CD8+ lymphocytes (CD8+ T cells and NK cells), rhesus macaques received a single dose of 50 mg/kg rhesus IgG1 recombinant Anti-CD8α monoclonal antibody MT807R1 (NIH NHP Reagent Resource) by intravenous route [61]. Absolute CD8+ T-cell counts (Ab clone, SK1 and RPA-T8, BioLegend, San Diego, CA) and viral loads were measured following MT807R1 infusion.

### 2.9. ELISA

ELISA plates were coated with AD8 gp120 at 5 μg/mL in PBS at 4 °C overnight. After blocking with 1% BSA in PBS at 37 °C for 1 h, serially diluted plasma was incubated on a plate at 37 °C for 1 h. 1:1000 diluted Horseradish peroxidase (HRP)-conjugated goat anti-human IgA + IgG + IgM (Jackson ImmunoResearch Laboratories, Inc., West Grove, PA) was added at 37 °C for 1 . All volumes were 100 μL/well, except 200 μL/well for blocking. Plates were washed between each step with 0.1% Tween 20 in PBS, developed with 3,3′,5,5′-tetramethylbenzidine (TMB) (Sigma-Aldrich, USA), and read at 450 nm.

### 2.10. ADCC Assay

ADCC assays were performed as previously described. Serum samples were serially diluted twice in AIM V™ medium (Gibco, Waltham, MA, USA) in 96-well V-bottom plates, starting at 1:30 and then 3-fold dilutions. An effector: target (E:T) ratio of 2:1 and an incubation time of 4 h. After the addition of Britelite Plus luciferase reporter reagent (PerkinElmer, Waltham, MA, USA), the plates were incubated for 5 min, and relative luminescence units (RLU) were read on a luminometer (TopCount NXT Luminescence Counter, PerkinElmer).

### 2.11. Statistical Analysis

Statistical analyses were performed by GraphPad Prism 10.3 Software (GraphPad Software, San Diego, CA, USA). A statistical comparison between groups at different time points during treatment was analyzed using the Wilcoxon rank sum test. A nominal α level of 0.05 was used to define statistical significance, and the data are presented as the mean and SEM.

## 3. Results

**Figure 1:**
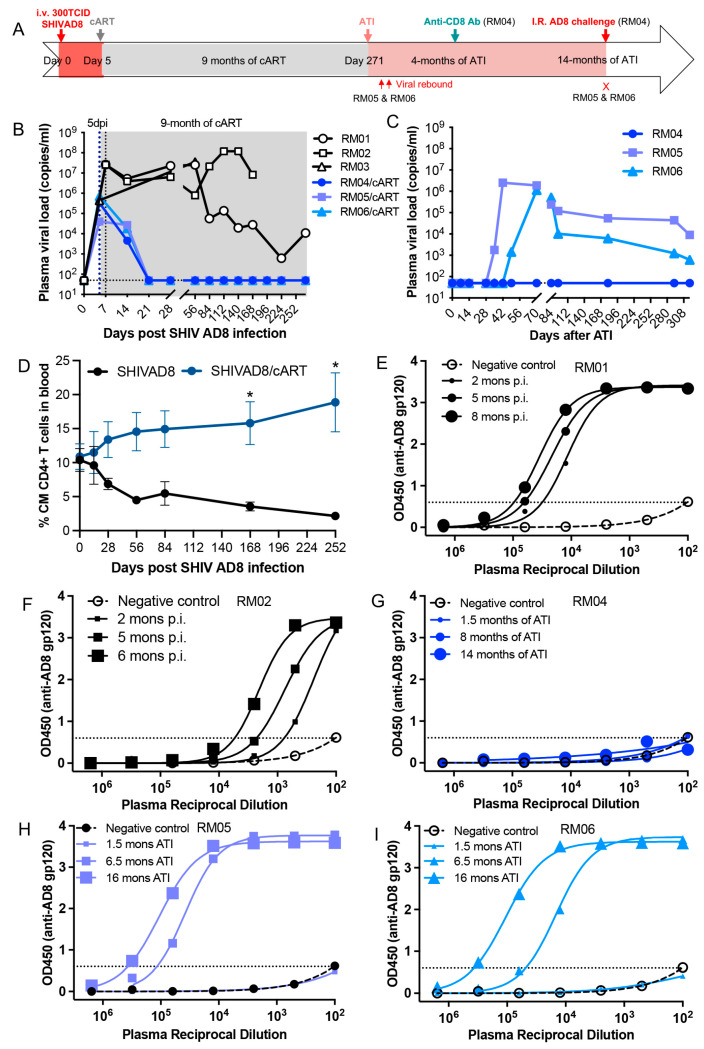
Efficacy of early antiretroviral therapy in suppressing viral replication and post-treatment viral recrudescence in SHIV-infected infant macaques following treatment initiation at 5 days post-infection. (**A**) Study outline for infant macaques intravenously inoculated with SHIV AD8, initiation of early retroviral therapy, and subsequent analytical treatment interruption. (**B**,**C**) Plasma viral load in infant cohorts receiving a 9-month course of cART initiated at 5 dpi and viral rebound after analytical treatment interruption (*n* = 3), compared to untreated controls (*n* = 3). (**D**) Changes in peripheral central memory (CM) CD4+ T cell subsets in treated infant macaques (colored line, *n* = 3) compared to age-matched untreated infant animals (black line, *n* = 3). Data are presented as mean ± SEM of % CM CD4+ T cells at each time point. *, *p* < 0.05 between animal cohorts at the same time point. (**E**–**I**). Plasma titers of anti-AD8 gp120 antibodies in individual infant animals at different time points, with (RM04, RM05, and RM06) or without (RM01, RM02) early treatment. Note that anti-AD8 gp120 antibodies were not detected in infant RM04 over time.

Delayed initiation of early antiretroviral therapy impacts virological outcomes in infant macaques infected with SHIV AD8 at birth. In our previous studies, we reported that 4 of 5 SIV-infected infant macaques achieved ART-free viral remission when early ART was initiated at 3 days post-infection (dpi) [45]. To investigate the effects of delayed early treatment on treatment outcomes, newborn macaques were intravenously inoculated with SHIV AD8 within 24 h of birth. cART was initiated at 5 dpi, a two-day delay compared to earlier studies, and continued for up to 9 months, with untreated animals serving as controls. Any infant macaque that exhibits viral remission following analytical treatment interruption (ATI) is subsequently administered anti-rhesus CD8 antibody (MT-807R1) to assess sustained virologic control (Figure 1A). As shown in Figure 1B, plasma viral load was consistently detectable at 5 dpi prior to cART initiation. After treatment began, viremia declined and became undetectable by day 21, remaining suppressed throughout the treatment period in treated animals, in contrast to untreated controls. However, two of the three SHIV AD8-infected, cART-treated infant macaques experienced viral rebound after ATI, on day 33 (RM05) and day 47 (RM06), respectively. Only one infant macaque (RM04) maintained an undetectable plasma viral load for up to approximately 14 months following treatment discontinuation (Figure 1C). Previously, we reported a progressive decline in peripheral central memory (CM) CD95 + CD28 + CD4+ T cells in untreated, SIV-infected infant macaques [62]. In contrast, early cART initiation at 5 dpi significantly preserved these memory CD4+ T cell subsets throughout the treatment course (Figure 1D). Given that seroconversion reflects the presence of anti-viral antibodies due to viral antigen exposure [63], we measured anti-AD8 gp120 antibody titers in individual infant macaques, including untreated controls and treated animals, at different time points after ATI. As shown in Figure 1E–I, anti-AD8 antibody titers correlated closely with detectable plasma viral load in both control animals and treated infant macaques who showed viral rebound post-ATI. Notably, anti-AD8 antibodies remained undetectable in infant macaque RM04, the only infant animal that achieved sustained ART-free viral remission (Figure 1G). These findings highlight the critical importance of timely treatment initiation in neonates exposed to HIV/SHIV for achieving sustained, ART-free virologic control.

**Figure 2:**
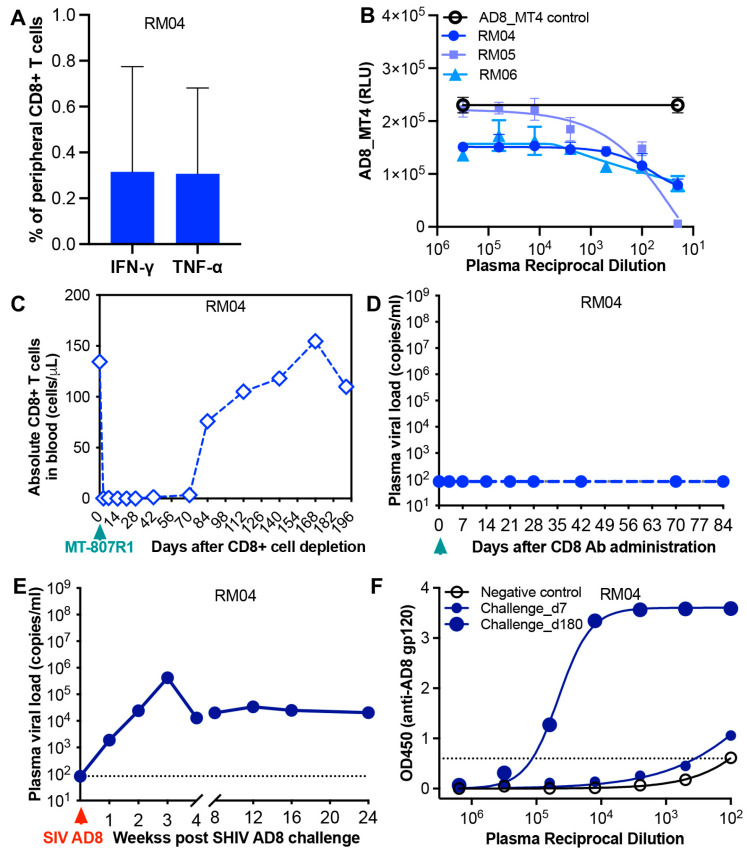
Virus-specific immunity in SHIV AD8-infected infant macaques, with or without initiation of treatment at various 5 days post-infection. (**A**) SIV gag-specific CD8+T cells in PBMCs isolated from virus-controlled RM04 at 4 months after treatment interruption. (**B**) Results of the ADCC assay for the 3 infant macaques receiving early treatment and 4 months after treatment discontinuation. Data are presented as the mean ± SEM of technical duplicates, representative of three independent experiments. (**C**) Absolute numbers of peripheral CD8+ T-cells in infant RM04 following intravenous anti-CD8α Ab administration at 4 months post-ATI. (**D**) Plasma viral load in RM04 over time during CD8+ cell depletion in vivo. (**E**) Plasma viral load in RM04 after receiving a single dose of 1000TCID50 SHIV AD8 via the intrarectal route at 14 months post-ATI. (**F**) Plasma titers of anti-AD8 gp120 antibodies in infant RM04 at day 7 and month 6 post SHIV AD8 challenge.

Immunological and virological determinants in achieving sustained viral remission in SHIV AD8-infected infant macaque treated at 5 days post-infection. To evaluate whether host immunity contributed to viral control in SHIV AD8-infected infant macaques, particularly in the infant that exhibited sustained virologic remission after ATI, we assessed multiple immunological parameters. First, we examined MHC class I alleles in the experimental macaques. Three alleles known to be protective (*Mamu-A01*, *Mamu-B08*, and Mamu-B*017) were generally absent, including in RM04, the infant that achieved sustained virologic remission post-ATI (Supplementary Table S1). This suggests that cytotoxic T lymphocyte (CTL) responses were unlikely to be responsible for viral control in this case. Specifically, SHIV Gag-specific CTL responses in RM04 were low or undetectable (Figure 2A). Next, we assessed plasma neutralizing antibodies (nAbs). As shown in Table 1, low titers of autologous nAbs were detected only in the untreated AD8-infected infant (RM01) at 6- and 8-months post-infection. nAbs were essentially absent in both treated and untreated animals. Notably, RM04, despite achieving viral remission off cART, had no detectable nAbs, consistent with the absence of total anti-AD8 Env antibodies (Figure 1G). We also evaluated antibody-dependent cellular cytotoxicity (ADCC) in the three cART-treated infants at 6 months post-ATI. Although RM05 and RM06 showed the presence of anti-AD8 Env antibodies, none of the animals exhibited detectable ADCC activity (Figure 2B). To determine whether RM04 had achieved sterilizing cure or simply durable remission, we depleted CD8^+^ T cells in vivo using the rhesus anti-CD8α antibody (MT-807R1) at 4 months post-ATI. As shown in Figure 2C, peripheral CD8^+^ cells were completely depleted for up to 70 days. Remarkably, no viral rebound or transient viremia (“blips”) occurred during this period (Figure 2D). We further analyzed cell-associated viral DNA in PBMCs, lymph node mononuclear cells (LNMCs), and rectal lymphocytes. In RM04, viral DNA remained undetectable during treatment and post-ATI, in contrast to detectable levels in LNMCs from RM05 and RM06, both of whom experienced viral rebound (Table 2). Finally, to assess whether RM04 had developed protective immunity, we performed a single high-dose intrarectal (IR) SHIV AD8 challenge at 14 months post-ATI. Plasma viremia became detectable one week post-challenge, indicating new infection (Figure 2E). This was supported by a subsequent rise in anti-AD8 Env antibody titers (Figure 2F). Collectively, these findings demonstrate that sustained virologic remission in RM04 was not mediated by virus-specific immune responses. Rather, it likely resulted from early and effective virologic suppression by antiretroviral therapy.

## 4. Discussion

Our recent study demonstrated that viral reservoirs in neonatal macaques exposed to SIV are not fully established by 3 days post-inoculation. Early cART initiation had markedly different effects on plasma viral load, even with just a one-day difference in timing (e.g., at 3, 4, or 5 dpi). Notably, a 9-month daily cART regimen initiated at 3 dpi resulted in sustained ART-free viral remission in approximately 4 out of 5 infant macaques. Additionally, infants living with HIV have been shown to develop broadly neutralizing antibodies (bnAbs) more rapidly than adults [8,9]. In this study, we investigated treatment outcomes in SHIV-exposed, infected infant macaques receiving a two-day delayed cART initiation (at 5 dpi), compared to outcomes observed with treatment at 3 dpi. We also assessed the induction of host immune responses under different conditions: prolonged viral exposure (untreated), transient viral suppression (treated with post-ATI viral rebound), and minimal to no antigen exposure (treated with sustained remission post-ATI). Among the treated animals, 1 out of 3 infant macaques achieved sustained virologic remission after treatment cessation. Notably, host immune responses, including virus-specific cytotoxic T lymphocytes (CTLs), antibody-dependent cellular cytotoxicity (ADCC), and neutralizing antibodies, were undetectable in this infant. This lack of immune correlates was further validated by the rapid acquisition of infection following an intrarectal SHIV challenge. These results suggest that viral control in this case was not immune-mediated, but rather due to the early virologic impact of antiretroviral therapy.

While the rate of pediatric viral remission dropped to 1 out of 3 when cART was initiated at 5 dpi, the timing of early treatment clearly influenced ART-free virologic control. This likely reflects differences in the establishment of early viral reservoirs in neonates, as shown in our previous work, indicating distinct dynamics in reservoir formation in SIV-exposed neonates compared to adults [5,6]. These findings underscore the importance of a narrow window for early pediatric intervention. Due to limitations in sample availability from surgical biopsies in neonatal and infant macaques, we were unable to comprehensively assess viral reservoirs across systemic and lymphoid tissues at 5 dpi prior to treatment, except in cases where necropsy was performed. However, recent findings from another study showed extensive seeding of viral reservoirs in various anatomical sites by 7 dpi in an SIV-exposed infant macaque. This is consistent with our observation that initiating treatment at 5 dpi failed to fully eliminate established viral reservoirs.

Findings utilizing pediatric NHP studies of HIV often appear inconsistent or even contradictory, likely due to variations in factors such as the inoculation route and the virus strains used. In neonatal macaques inoculated intravenously (IV) with SIV, plasma viral load and cellular SIV RNA/DNA are readily detectable as early as 1 dpi and beyond [45]. In contrast, studies involving oral HIV/SIV exposure in infants often report undetectable viremia during the initial days of infection, with cell-associated viral RNA/DNA detected only sporadically in certain tissues during early infection stages [36,37,38,39,40,41,42,43,44]. It remains unclear whether these discrepancies arise from differing experimental conditions or variations in assay sensitivity. Given that SIVmac is highly pathogenic and possesses greater infectivity compared to the less pathogenic SHIV strains [64], orally SHIV-exposed infants, such as those infected with SHIV162p3 or SHIV AD8, likely exhibit distinct patterns of early viral reservoir establishment. For instance, in infant macaques orally inoculated with SHIVSF162P3, bnAb therapy administered at 30 h post-inoculation, or a 21-day cART regimen initiated at 48 h, was shown to clear tissue viral reservoirs [37,38]. In contrast, our studies revealed that unintegrated viral DNA remained detectable in PBMCs and/or LNMCs even after 28 days in some IV SIV-infected infants receiving cART at 2 dpi. These findings suggest that viral pathogenicity significantly influences tissue distribution and the susceptibility of viral reservoirs to antiretroviral therapy. Moving forward, future studies should prioritize mucosal transmission models using currently available transmitted/founder SHIV strains to better reflect the dynamics of human perinatal HIV infection.

Fundamental gaps remain in our understanding of pediatric HIV infection and the effectiveness of early treatment. The variability in viral reservoir eradication and differing outcomes observed following early intervention in infants are likely attributable to factors such as immune system maturation, developmental stage, host susceptibility, genetic background, and maternal influences [6]. It has been proposed that the rate of initial viral suppression correlates with the likelihood of achieving HIV remission [65]. The distribution and size of early viral reservoirs may significantly influence the rate of plasma viral load decline in infant macaques receiving early cART, ultimately impacting treatment outcomes. Based on our previous and current studies in SIV/SHIV-exposed neonatal macaques, achieving a treatment-free functional cure appears more likely when viral replication is rapidly and efficiently suppressed, specifically, when plasma viral load becomes undetectable by day 7 post-infection. In contrast, delayed suppression, such as undetectable viral load achieved by 14 dpi or later, is associated with a higher likelihood of viral rebound following treatment interruption, as demonstrated in a substantial number of infant macaques in our studies. Additionally, SHIV AD8-infected infant macaques failed to develop potent bnAbs, despite exhibiting high titers of total anti-AD8 Env antibodies, which contrasts with the bnAb responses observed in adult macaques [53]. The absence of bnAb induction in ART-treated infants is expected, given their minimal to absent viral antigen exposure during treatment. Another contributing factor may be the relatively brief duration of Env exposure (less than one year) in both untreated infants and those who experienced post-treatment viral rebound [66]. Importantly, neither antibody- nor cell-mediated immune responses were detected in early-treated infant macaques that achieved ART-free viral remission, suggesting that virologic control and reservoir elimination were primarily driven by the direct antiviral effects of cART. Overall, this study evaluates the impact of a two-day delay in treatment initiation compared to our earlier findings, providing new insights into the critical role of early pediatric intervention and its implications for clinical strategies aimed at achieving long-term HIV remission in infants.

## Figures and Tables

**Table 1:** Plasma neutralizing antibodies.

Animal ID	Months p.i.	Status	Neutralizing Antibody Titers	
			SHIVCL7V3AD8	SHIVAD8EO
RM01	3	No cART	1:51	<1:20
	6	No cART	1:312	<1:20
	8	No cART	1:313	<1:20
RM02	3	No cART	<1:20	<1:20
	6	No cART	<1:20	<1:20
RM03	2	No cART	<1:20	<1:20
RM04	12	3 mons ATI	<1:20	<1:20
RM05	12	3 mons ATI	1:36	<1:20
RM06	12	3 mons ATI	<1:20	<1:20

**Table 2:** Detection of cell-associated SHIVAD8 DNA in samples (copies per million cells).

Animal ID	Sample	6 Months of cART	8 Months of ATI	14 Months of ATI
RM04	PBMCs	<20	<20	<20
	LNMCs	<12	<9	<5
	Rec lymphocytes	NA	<6	NA
RM05	LNMCs	NA	NA	1.52 × 10^3^
RM06	LNMCs	NA	NA	5.41 × 10^2^

LNMCs, lymph node-derived mononuclear cells; NA, not applicable.

## Data Availability

All data reported in this paper will be shared by the correspondence author upon request.

## Supplementary Materials

The following supporting information can be downloaded at: https://www.mdpi.com/article/10.3390/v17060849/s1. Table S1. MHC I alleles in the six experimental infant macaques; Table S2. Primers and hydrolysis probe sequences for gene targets.
