# Supplementary material for: Impact of Delayed Early Antiretroviral Therapy Initiation on Treatment Outcomes in Infant Macaques Exposed to SHIVAD8
Source: Viruses. 2025 Jun 14;17(6):849. doi: 10.3390/v17060849 (PMC12197331; doi:10.3390/v17060849)
Supplement: Supplementary file 1 [file viruses-17-00849-s001.zip › viruses-3618582-supplementary.pdf]

Table S1. MHC I alleles in the six experimental infant macaques.

Animal ID	A*01	A*02	A*08	A*11	B*01	B*03	B*04	B*08	B*17	DRB*w201
POS	+	+	+	+	+	+	+	+	+	+
NEG	−	−	−	−	−	−	−	−	−	−
RM01	−	+	−	−	−	−	−	−	−	+
RM02	−	−	−	+	−	−	−	−	−	+
RM03	−	−	−	−	−	−	−	−	−	−
RM04	−	+	+	−	−	−	−	−	−	+
RM05	−	−	+	−	−	−	−	−	−	−
RM06	+	−	−	−	−	−	−	−	−	−

Table S2. Primers and hydrolysis probe sequences for gene targets.

Target	Oligonucleotide	Sequence (‘5 to 3′)	Amplicon Length
	Forward primer	TCAGCATGGCGGTGTTT	
RPPR30	Reverse primer	GCTGTCTCCACAAGTC	48bp
	Probe	(VIC)-TTCTGACCTGAAGGCTCTGCGC- (3IABkFQ-1))	
	Forward primer	GTCTGCGTCATCTGGTGCATTC	
SIV Gag	Reverse primer	CACTAGGTGTCTCTGCACTATCTGTTTTG	60bp
	Probe	(FAM)-CTTCCTCAGTGTGTTTCACTTTCTCTTCTGCG-(3IABKFQ-1)	
